# ^18^F-FDG PET-CT pattern in idiopathic normal pressure hydrocephalus

**DOI:** 10.1016/j.nicl.2018.02.031

**Published:** 2018-02-28

**Authors:** Ryan A. Townley, Hugo Botha, Jonathan Graff-Radford, Bradley F. Boeve, Ronald C. Petersen, Matthew L. Senjem, David S. Knopman, Val Lowe, Clifford R. Jack, David T. Jones

**Affiliations:** a Department of Neurology Mayo Clinic, Rochester, MN 55901, USA; b Department of Diagnostic Radiology, Mayo Clinic, Rochester, MN 55901, USA; c Information Technology Radiology, Mayo Clinic, Rochester, MN 55901, USA

**Keywords:** FDG-PET, Normal pressure hydrocephalus, Hypometabolism, Caudate, Biomarker

## Abstract

**Background:**

Idiopathic normal pressure hydrocephalus (iNPH) is an important and treatable cause of neurologic impairment. Diagnosis is complicated due to symptoms overlapping with other age related disorders. The pathophysiology underlying iNPH is not well understood. We explored FDG-PET abnormalities in iNPH patients in order to determine if FDG-PET may serve as a biomarker to differentiate iNPH from common neurodegenerative disorders.

**Methods:**

We retrospectively compared ^18^F-FDG PET-CT imaging patterns from seven iNPH patients (mean age 74 ± 6 years) to age and sex matched controls, as well as patients diagnosed with clinical Alzheimer's disease dementia (AD), Dementia with Lewy Bodies (DLB) and Parkinson's Disease Dementia (PDD), and behavioral variant frontotemporal dementia (bvFTD). Partial volume corrected and uncorrected images were reviewed separately.

**Results:**

Patients with iNPH, when compared to controls, AD, DLB/PDD, and bvFTD, had significant regional hypometabolism in the dorsal striatum, involving the caudate and putamen bilaterally. These results remained highly significant after partial volume correction.

**Conclusions:**

In this study, we report a FDG-PET pattern of hypometabolism in iNPH involving the caudate and putamen with preserved cortical metabolism. This pattern may differentiate iNPH from degenerative diseases and has the potential to serve as a biomarker for iNPH in future studies. These findings also further our understanding of the pathophysiology underlying the iNPH clinical presentation.

## Highlights

• The pathophysiology of iNPH is poorly understood.
• Clinical biomarkers for diagnosis and prediction of shunt outcome are needed.
• iNPH patients have striatal hypometabolism on FDG-PET.
• Striatal hypometabolism helps explain the clinical presentation of iNPH.
• FDG-PET is a useful tool for differentiating iNPH from other degenerative disorders.

## 1 Introduction

Idiopathic normal pressure hydrocephalus (iNPH) is a treatable neurologic syndrome which has become more relevant in recent years due to an aging population. The hallmark clinical features of iNPH are gait and balance dysfunction, urinary symptoms, and cognitive impairment, occurring in the setting of ventriculomegaly on neuroimaging and in the absence of elevated cerebrospinal fluid (CSF) pressure. Problems with gait, urinary incontinence, and cognitive impairment occur more commonly with increasing age – ranging from 15 to 42% depending on the population studied (Coyne et al., 2014; Petersen et al., 2014; Sudarsky, 1990). This complicates the diagnosis of iNPH, the treatment approach, and outcome measurements. A recent epidemiology study suggested that iNPH may be underdiagnosed, particularly in assisted-living or extended-care residents (Martin-Laez et al., 2015).

Treatment of iNPH is invasive and not without risk, but with appropriate patient selection and early intervention, a CSF diverting shunt can improve symptoms in 60–80% of patients (Duinkerke et al., 2004; Poca et al., 2012). Time to intervention and proper patient selection are the most important determinants in overall postoperative improvement rates (McGirt et al., 2005). Thus, research has focused on improving diagnostic accuracy and predicting shunt response. Patients are selected following a multimodal, staged evaluation which includes clinical examination, neuroimaging, and often a large volume spinal tap. This process is complicated by the fact that iNPH can be difficult to differentiate from other neurodegenerative diseases causing ventriculomegaly.

Memory impairment may be misdiagnosed as early Alzheimer disease dementia (AD). Subcortical attentional difficulties and executive dysfunction can mimic Dementia with Lewy bodies (DLB) or Parkinson Disease Dementia (PDD). Gait abnormalities, postural instability, and imbalance can mimic parkinsonian syndromes. Apathy and personality changes can mimic early behavioral variant frontotemporal dementia (bvFTD). To complicate matters further, iNPH patients can have co-existing neurodegenerative diseases due to age related factors (Cabral et al., 2011, Golomb et al., 2000).

18-F-fluorodeoxyglucose positron emission tomography/computed tomography (FDG-PET) reliably distinguishes common neurodegenerative diseases from control patients (Silverman, 2004). FDG-PET was first described as differentiating AD and NPH in 1985 with three patients by demonstrating an absence of an AD pattern in NPH rather than a pattern unique to NPH (Jagust et al., 1985). A unique pattern of hypometabolism has not been described in iNPH, and to our knowledge, FDG-PET has not been assessed to differentiate iNPH from other degenerative disorders. We aimed to address this knowledge gap by retrospectively evaluating the FDG-PET findings in cases of iNPH and comparing this to controls and common neurodegenerative disorders.

## 2 Methods

### 2.1 Patient selection

Design and implementation of this single-center retrospective study met HIPAA guidelines and was approved by the Mayo Clinic Institutional Review Board. Minimal risk criteria were met and need for informed consent was waived. We searched the Mayo Clinic database for ICD9 codes 331.3, 331.4, and 331.5 (Hydrocephalus) and identified patients who had FDG-PET and MR brain imaging performed as part of their diagnostic evaluation using CPT codes. A chart review was performed on these patients who had FDG-PET imaging performed and met our inclusion criteria: probable clinical diagnosis (Williams and Relkin, 2013), supportive MRI brain findings (Hashimoto et al., 2010), positive response to lumbar puncture, and FDG-PET imaging absent of another neurodegenerative pattern to explain symptoms.

To compare iNPH patients to degenerative disorders we randomly selected age and sex matched patients from the Mayo Clinic Alzheimer's Disease Research Center (ADRC) meeting clinical criteria for AD (matched 2:1), DLB/PDD (matched 2:1) or bvFTD (matched 1:1). All diagnostic groups were compared to cognitively normal controls from the Mayo Clinic Study of Aging (Roberts et al., 2008). The control group was also randomly age and sex matched (3:1) to the iNPH group. All ADRC participants or their designees provided written consent with approval of the Olmsted Medical Center and/or Mayo Clinic Institutional Review Boards.

### 2.2 Image acquisition

All patients had FDG-PET images acquired using a PET/CT scanner (GE Healthcare) operating in 3D mode. Patients were injected with 18-F-fluorodeoxyglucose in a dimly lit room, and after a 30 min uptake period an 8-min FDG scan was performed, which consisted of four 2-min dynamic frames following a low dose CT transmission scan. All patients also had 3.0 T MR imaging which included a 3-D MPRAGE sequence.

### 2.3 Image processing

All MRI were spatially normalized and FDG-PET images were co-registered to each patient's MRI using unified segmentation and normalization procedure in SPM12 (http://www.fil.ion.ucl.ac.uk/spm/software/spm12/). The pons was identified after propagating the automated anatomical labeling atlas to native MRI space. Intensity was normalized using pons as the reference and smoothed with an 8 mm full-width half maximum Gaussian kernel. Next, FDG-PET voxels were divided by the median uptake in the pons. The resulting FDG uptake ratio images were then normalized to the custom template using the parameters from MRI normalization. The structural MRI was used to perform a partial volume correction (PVC) for tissue and CSF compartments to mitigate partial volume confounding contributions to the observed decreased FDG-PET signal (Meltzer et al., 1999).

### 2.4 Statistical analysis

Analysis of variance (ANOVA) statistical methods were used for age and MRI-PET date range across groups. To identify patterns of hypometabolism between groups, we utilized SPM12 running on MATLAB R2015b (Mathworks, Natick, MA). Each diagnostic group was compared to controls in SPM12 using a one-way *t*-test and to each other with two-way *t*-tests (Ashburner, 2009). Results were viewed after *p* < 0.05 family wise error correction (FWE) was applied at the cluster level, using a height threshold of 5.57 and extent threshold k of 40 as determined with REST AlphaSim (Song et al., 2011). MRICron and BrainNet Viewer were used to present results (Rorden and Brett, 2000; Xia et al., 2013). The SPM ‘Anatomy toolbox’ was used to label areas of maximal hypometabolism (Eickhoff et al., 2005). In a post-hoc analysis, the median metabolism in the striatum was determined for each patient using the caudate and putamen ROIs from the AAL atlas (Tzourio-Mazoyer et al., 2002). Group statistical comparisons of striatum metabolism were then performed using a one-way ANOVA test with Tukey HSD post hoc pairwise analysis to compare the iNPH group to controls, AD, DLB/PDD, and bvFTD groups.

## 3 Results

Seven patients met our inclusion criteria. The average age of the iNPH group was 74.3 years old, and the average Short Test of Mental Status (STMS) was 24.8/38 (Tang-Wai et al., 2003). Four of the seven iNPH patients received a shunt at Mayo Clinic and all had significant clinical improvement. The other three patients returned closer to home for shunt placement or were lost to follow up. Demographics are shown in Table 1. Notably, bvFTD patients were significantly younger than other groups, including iNPH patients.

**Table 1:** Group demographics comparison.

	Controls	AD	DLB/PDD	bvFTD	iNPH	*p*-Value
# of patients	21	14	14	7	7	
Age	74.3	74.3	74.3	66	74.3	<0.01
Sx duration (years)	N/A	4.8	6	5.8	3.1	0.12
STMS⁎⁎	N/A	27.3	24.7	21.6	24.8	0.44
MRI-PET difference, Mean ± SD, days⁎	21 ± 20	15 ± 35	27 ± 40	1 ± 1	38 ± 70	0.33

⁎ The MRI-PET difference (in days) describes the average amount of days between the MRI acquisitions that were used to coregister with the FDG-PET scan.

⁎⁎ Short Test of Mental Status.

**Fig. 1:**
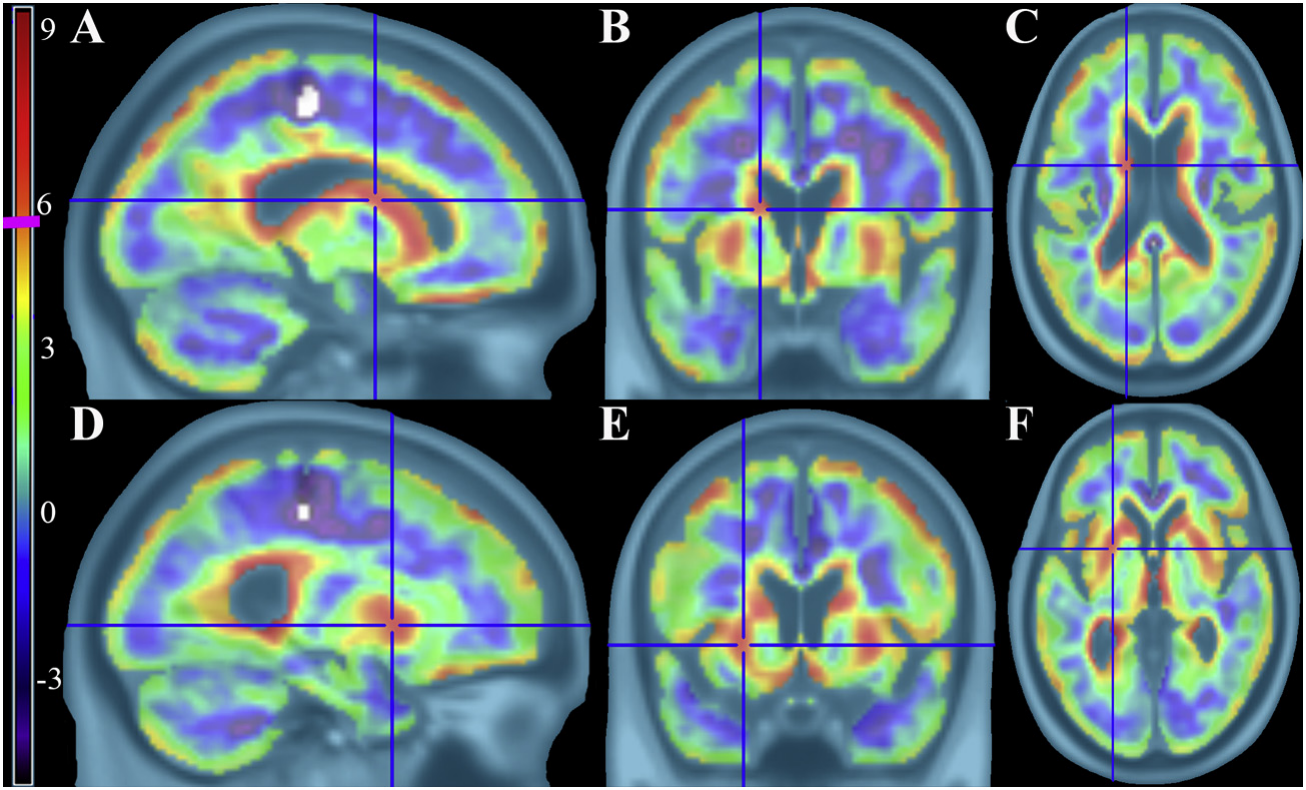
Controls > iNPH t-maps using non-PVC corrected FDG-PET images showing significant hypometabolism in iNPH patients. There is clear volume averaging artifact around the ventricles and cortical surface. Vertical t-score bar on the left with pink line at significance height threshold of 5.57 ((*p* < 0.05, FWE corrected, k = 40) A–C) Left body of caudate in sagittal, coronal, and axial views D–F) Left putamen in sagittal, coronal, and axial views.

**Fig. 2:**
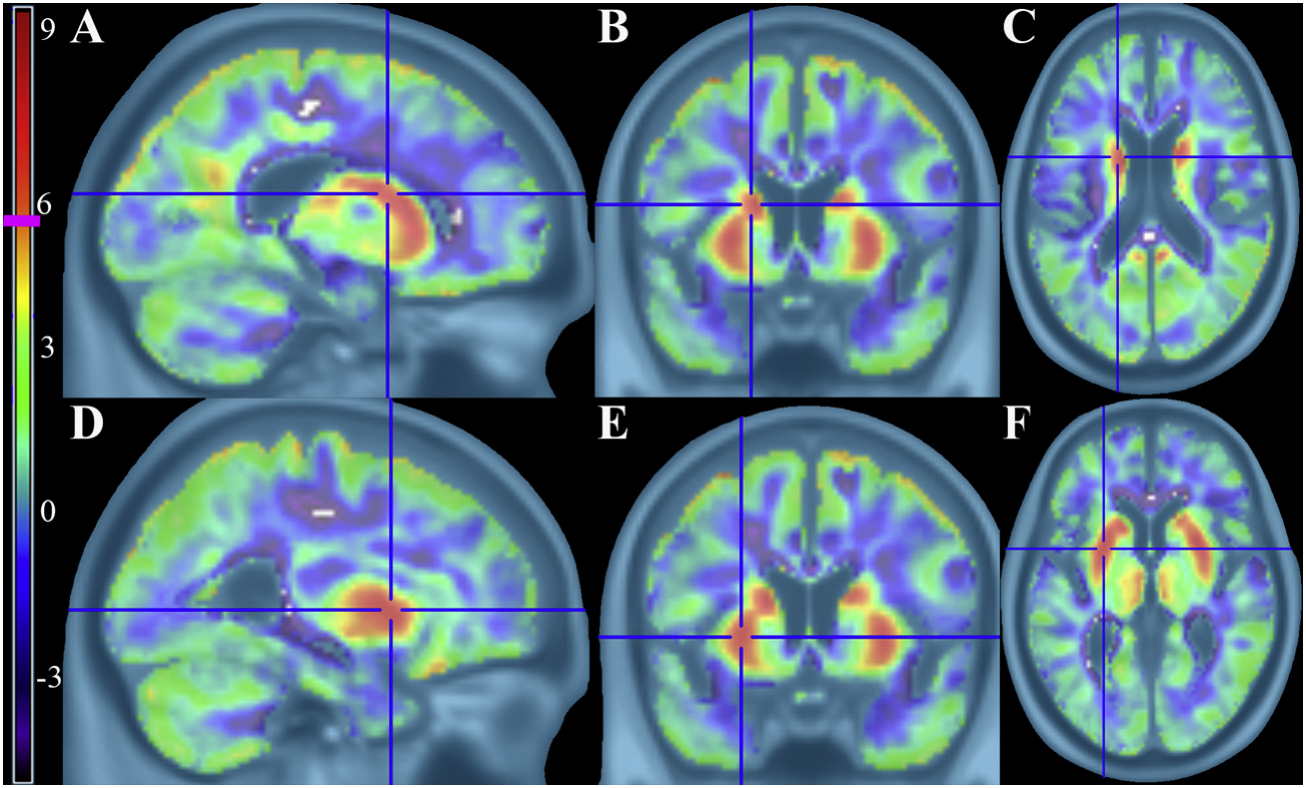
Controls > iNPH t-maps using PVC corrected FDG-PET images showing significant hypometabolism in iNPH patients. The volume averaging confound is no longer present, but the subcortical hypometabolism is clearly evident. Vertical t-score bar on the left with pink line at significance height threshold of 5.57 ((*p* < 0.05, FWE corrected, k = 40) A–C) Left body of caudate in sagittal, coronal, and axial views (peak t-score 8.01) D–F) Left putamen in sagittal, coronal, and axial views (peak t-score 7.42).

Results for the non-PVC comparison between controls and iNPH are shown in Fig. 1. The most significant differences were found in the bilateral dorsal striatum (caudate and putamen). These results survived PVC with high statistical significance, as shown in Fig. 2.

**Fig. 3:**
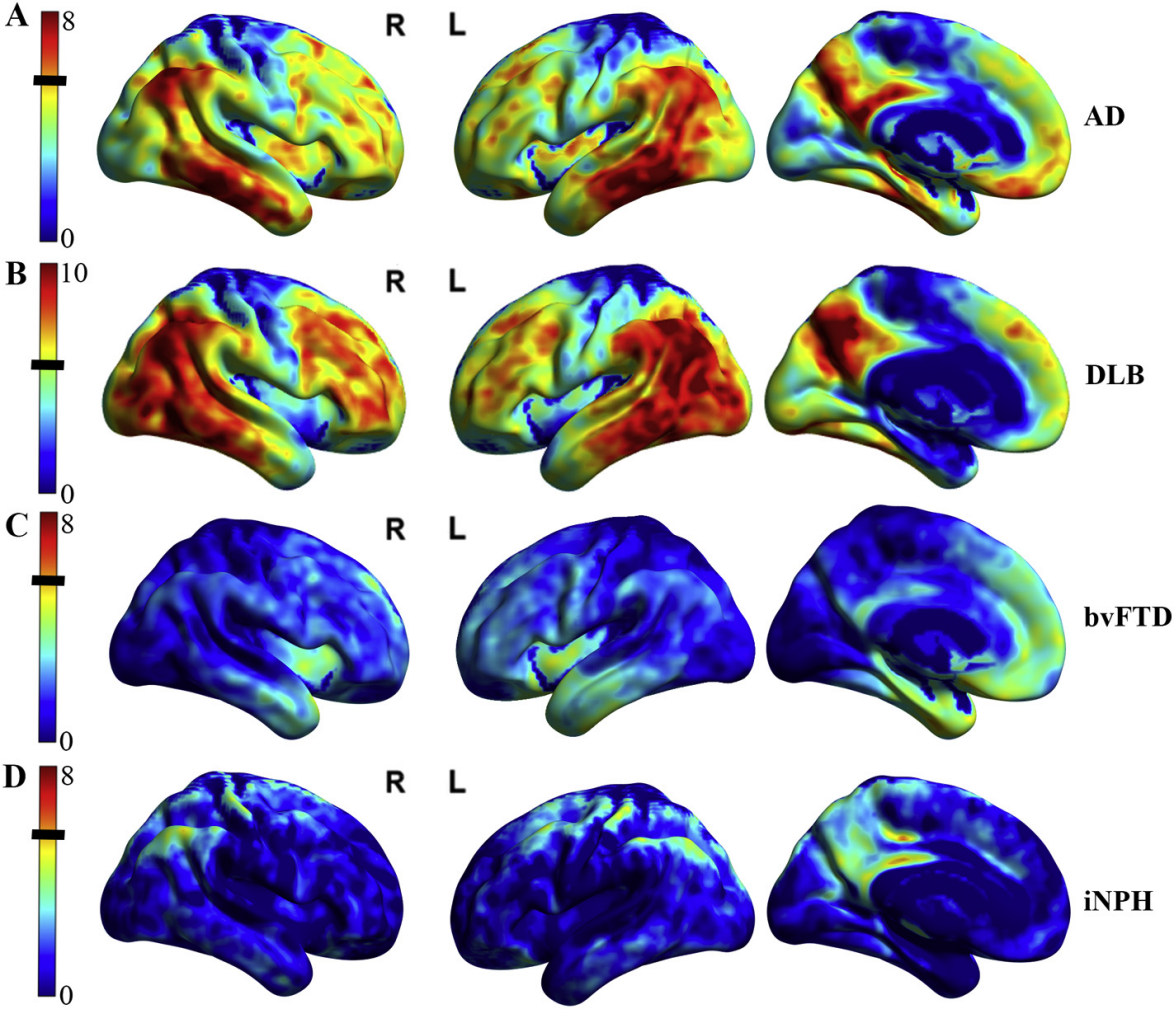
Cortical hypometabolic patterns across other disease groups compared to controls are displayed on surface renderings. Subcortical patterns are not visualized here. Height threshold t-bar on the left with black line indicating significance of *p* < 0.05 (FWE corrected, k = 40). A) AD vs. Control group showing predominantly temporoparietal, precuneus, and posterior cingulate cortex hypometabolism pattern. There was no significant caudate or putamen hypometabolism. B) DLB/PDD vs. Control group showing predominantly occipital, parietal, temporal, and precuneus (posterior cingulate spared) hypometabolism pattern. DLB/PDD had the greatest levels of hypometabolism across the groups and needed a larger dynamic range to visually capture this. There was also significant caudate hypometabolism with peak t value of 7.02 which is not visualized on these surface renderings. C) bvFTD vs. Control group showing lower level of significance of left > right fusiform gyrus, insula, and anterior cingulate cortex hypometabolism pattern. D) iNPH vs. Control group showing no specific pattern of significant cortical hypometabolism.

Compared to controls, patients with AD, DLB/PDD, and bvFTD showed expected patterns of cortical hypometabolism, shown in Fig. 3. In contrast, iNPH patients did not show a cortical hypometabolic pattern to account for their neurologic symptoms.

**Fig. 4:**
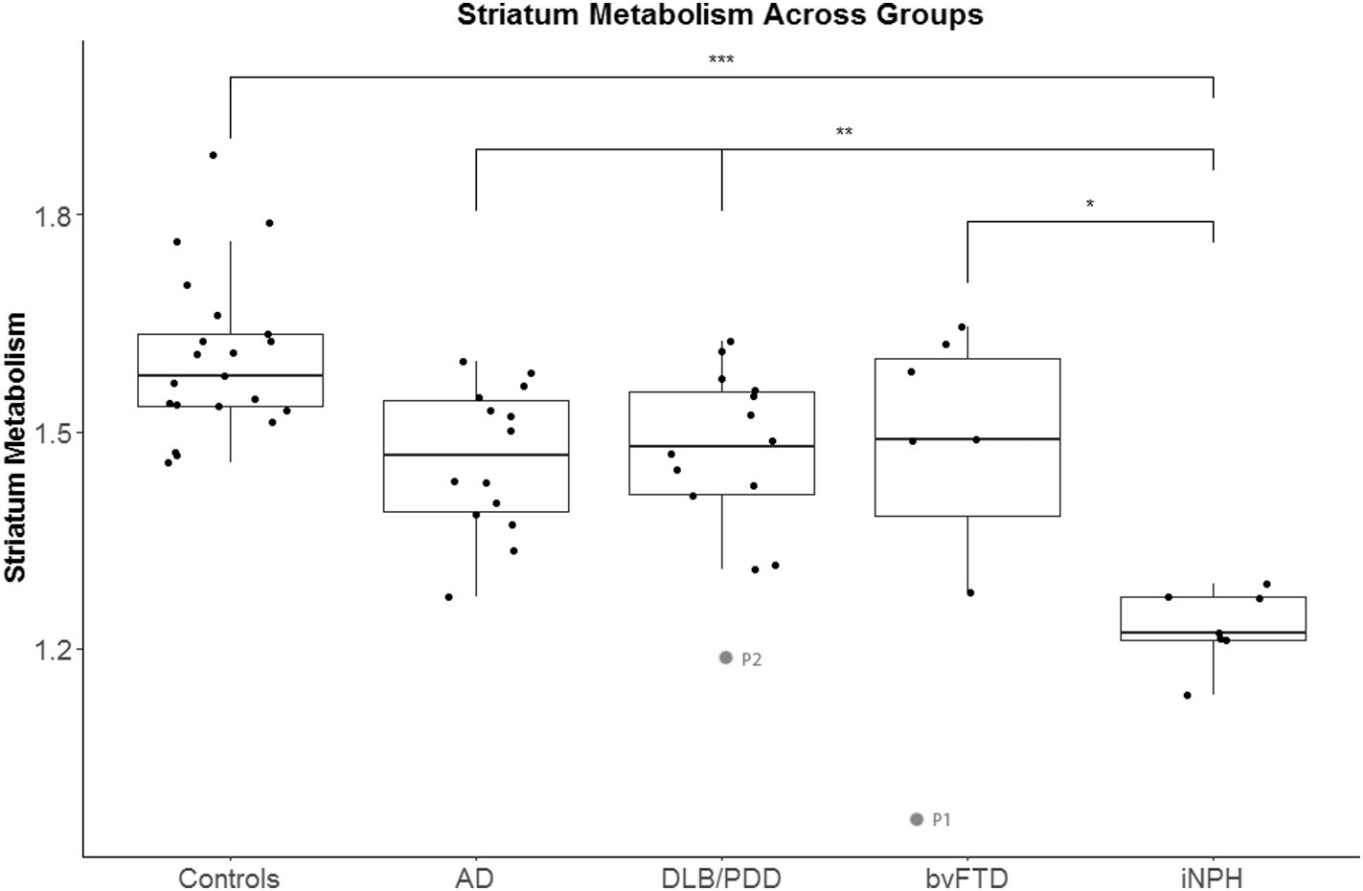
Box plot comparing striatum metabolism (a standardized ratio to the pons) across diagnostic groups. Area within the box indicates the 25th–75th percentile, the line within the box the median, the line outside the box the range between 1.5 standard deviations of the mean, and the solid circles indicate the individual cases for each group. The outliers are labeled P1 and P2 and included in the analysis. They are discussed in detail in the supplemental material. ^⁎⁎⁎^indicates highest significance CN > NPH (*p* < 0.0001). ^⁎⁎^Indicates high significance with DLB/PDD > NPH (*p* = 0.0020), AD > NPH (*p* = 0.0022). ^⁎^indicates moderate significance, FTD > NPH (*p* = 0.0387).

The results of the ROI-based analysis are shown in Fig. 4. When iNPH patients were compared to controls, striatal hypometabolism was highly significant (*p* < 0.0001). When iNPH patients were compared to AD and DLB/PDD groups, the differences in striatum hypometabolism were highly significant (*p* < 0.003). When compared to bvFTD patients the findings were significant (*p* < 0.04). There was a significant outlier in the bvFTD group with a progranulin gene mutation, labeled P1 on Fig. 4. Another outlier in the DLB/PDD group, labeled P2 on Fig. 4, had multiple pathologies on autopsy. These two outliers were included in the data analysis and are discussed in detail in the supplemental material and supplemental Figs. 1 and 2.

## 4 Discussion

Patients with iNPH had a pattern of subcortical hypometabolism, including bilateral dorsal striatum, which survived correction for partial voluming errors. The lack of cortical hypometabolism in our iNPH group suggests their cognitive and gait abnormalities may derive from striatal dysfunction rather than widespread cortical dysfunction or coexisting degenerative conditions. These results help further the understanding of iNPH pathophysiology, and serves as a potential biomarker for identifying proper shunt candidates. Future studies based on this original study could assess if striatum hypometabolism is reversible with shunt placement, and if the severity of striatum involvement predicts shunt outcome.

Previous FDG-PET studies have suggested that iNPH is a global brain disease, as evidenced by heterogeneous patterns and a global reduction in cerebral metabolic rate of glucose (CMRglu) compared to controls (Calcagni et al., 2013; Tedeschi et al., 1995). Our results differ from those studies, potentially due to improved partial volume correction techniques with co-registered MRI and due to patient selection, reducing the likelihood of coexisting degenerative disease. Our results concur with older cerebral blood flow (CBF) studies demonstrating decreased CBF in the thalamus, caudate, and putamen in iNPH (Owler et al., 2004). Previous MRI VBM studies found decreased caudate volumes in iNPH compared to controls (DeVito et al., 2007). More recent imaging techniques, such as CT perfusion (Ziegelitz et al., 2016), arterial spin-labeling MR perfusion (Virhammar et al., 2017), and dynamic susceptibility contrast-enhanced MR perfusion imaging (Tuniz et al., 2017) support our findings of changes in the dorsal striatum. These studies all found basal ganglia or lentiform nucleus predominant changes. To our knowledge, no one has compared iNPH FDG-PET to other degenerative diseases using voxel based analysis.

The neuropsychological profile of iNPH can be heterogeneous, but it consistently involves a frontosubcortical pattern of psychomotor slowing, gait apraxia, postural abnormalities, and executive dysfunction (Devito et al., 2005). Striatal dysfunction may underlie several of these deficits. Normal caudate function correlates with verbal fluency (Lawrence et al., 1998), executive function (Rogers et al., 2000), body and limb posture (Villablanca, 2010), and psychomotor speed.(Volkow et al., 1998). The putamen is important for automatic motor movements via projections from the motor cortex (Utter and Basso, 2008). There are many striatal interneuron connections between the putamen and caudate as well. The corticostriatal circuits have reciprocal communication to the supplemental motor cortex; an area crucial for postural stability, locomotion, and coordinating movements (Utter and Basso, 2008).

The caudate, located adjacent to dilating ventricles, may be the first affected in iNPH, as evidenced by reduced blood flow in prior studies and reduced metabolism in this study. Alternatively, it is possible that frontosubcortical circuits are dysfunctional, and this diffuse circuitry dysfunction most reliably manifests in focal alterations of metabolic function in the striatum. Highly connected striatal structures and more diffuse cortical structures may be affected later as the caudate nuclei, the thalamus, and their periventricular white matter connections are further compromised. This hypothesis is consistent with the findings describing duration of disease in iNPH patients being the major predictor of global cognitive impairment compared to limited frontosubcortical dysfunction (Picascia et al., 2016). Projecting white matter alterations are abnormal in diffusion tensor imaging in iNPH, compared to controls, AD, and PD subjects (Hattori et al., 2011). Recent studies have shown reduced cerebral blood flow in the lentiform nucleus and projecting periventricular white matter connections in iNPH patients and this reduced perfusion improved in shunt responders with no significant change in perfusion in non-responders (Ziegelitz et al., 2016). Our patients all had positive responses to lumbar puncture and those who were shunted had clinical improvement. Determining the timeline regarding specific biomarkers relation to reversible iNPH changes compared to non-reversible changes remains an important area of continuing study.

While other methods exist in evaluating for co-existing neurodegenerative disease, FDG-PET may provide a unique means to simultaneously evaluate for the presence of an iNPH specific pattern and the absence of other neurodegenerative diseases that commonly occur in older populations. As described in the current study, a subcortical pattern of hypometabolism with preserved cortical metabolism may improve identification of ideal shunt candidates and rule out co-existing degenerative etiologies that could portend poor clinical outcomes. For example, a cortical hypometabolic pattern consistent with DLB may predict a poor outcome to surgical intervention whether there is co-existing iNPH or not. In the right clinical context, an absence of cortical hypometabolism may be a positive prognostic marker in the setting of subcortical hypometabolism.

**Fig. 5:**
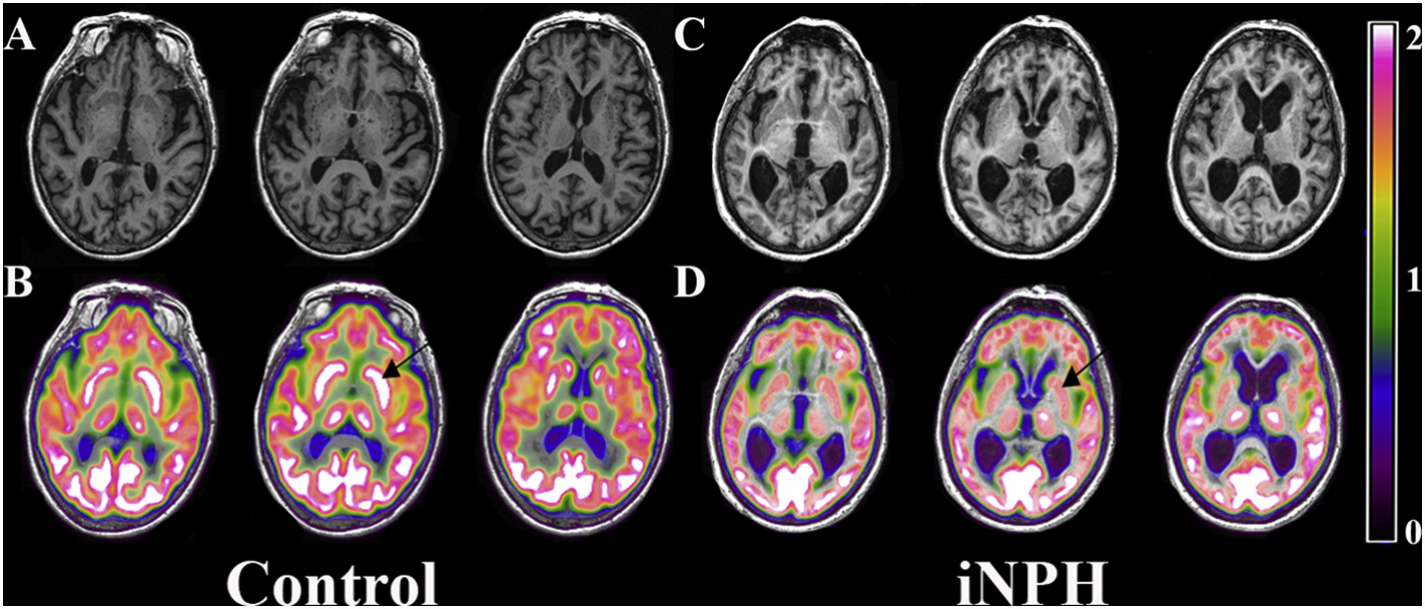
These images are in native patient space before warping to standard space for the group comparison. A) Shows a control patient with 3T MPRAGE MRI in axial slices. B) MRI with overlying normal FDG metabolism of the dorsal striatum (arrow points to left caudate). This is contrasted to C) an iNPH patient with 3T MPRAGE MRI in axial slices. D) MRI with overlying FDG metabolism depicting clear hypometabolism of the dorsal striatum (arrow points to left caudate). This figure provides further evidence that the caudate hypometabolism is not a registration error with the ventriculomegaly of an iNPH patient.

There are limitations to this study. To ensure accurate and isolated iNPH diagnosis, the inclusion criteria limited the sample size. Inclusion criteria were designed to try and avoid comorbidities, and this reduces the generalizability for all iNPH patients. This does not negate the fact that this pattern exists and may further help explain the clinical findings in iNPH. The caudate region, and in particular the body of the caudate, is also a small structure and one has to take caution with voxel based data with close proximity to enlarged ventricles, (Rahmim et al., 2013). However, appropriate steps were taken to exclude the possibility that hypometabolism was caused by volume averaging. The hypometabolism is also throughout the entire dorsal striatum and not just the periventricular regions. The results were also bolstered by the post hoc, ROI-based analysis of striatal metabolism. Additionally, Fig. 5 shows an example iNPH patient depicting the caudate hypometabolism accurately registering despite the ventriculomegaly. This was a retrospective pilot study and as a result many variables could not be controlled for. The time between CT/PET and MRI was not statistically significant between groups, but remains a source of potential error if pathology were to change between the two scans.

## 5 Conclusion

In summary, we present a subcortical FDG-PET pattern in iNPH that may differentiate it from age related changes in gait, bladder function and cognition. Despite cognitive tests in the abnormal range of other neurodegenerative diseases, the cortical metabolism in this subset of iNPH patients is not significantly different from control patients. The frontosubcortical dementia pattern in iNPH patients correlates well with these dorsal striatum hypometabolism findings. Whether or not FDG-PET can serve as a biomarker for disease progression and response to intervention remains to be seen. Future studies should prospectively enroll a larger group of patients with iNPH and analyze PVC corrected FDG-PET imaging before and after shunting. In doing so, FDG-PET can be properly incorporated into the diagnostic and prognostic process, hopefully resulting in earlier intervention in appropriately selected patients.

## Appendix A Supplementary data

Supplementary material. Image 1
