# Supplementary material for: 18F-FDG PET-CT pattern in idiopathic normal pressure hydrocephalus
Source: Neuroimage Clin. 2018 Feb 28;18:897–902. doi: 10.1016/j.nicl.2018.02.031 (PMC5987871; doi:10.1016/j.nicl.2018.02.031)
Supplement: Supplementary file 1 — Supplementary material. [file mmc1.docx]

Supplemental Material:

**Patient 1, labeled P1 on Figure 4:** This patient presented as a bvFTD phenotype and was later diagnosed with a *Progranulin* gene mutation. On further analysis, his FDG-PET scan shows diffuse hypometabolism, including severe caudate and putamen hypometabolism, shown in Supplemental Figure 1A. Their neuropsychometric testing at the time of the FDG-PET scan was unattainable due to severe dementia, clinically documented as 0/38 on the STMS. This case was included in the data analysis but is certainly an outlier and would not clinically be mistaken for iNPH.

**Patient 2, labeled P2 on Figure 4:** This patient was initially evaluated at Mayo Clinic for iNPH due to ventriculomegaly, cognitive problems and an abnormal gait. She had signs of early Parkinsonism on exam and a large volume lumbar puncture was minimally beneficial. The patient elected against shunt surgery at that time. In subsequent followups over 10 years at our ADRC, the patient’s Parkinsonism progressed and visual hallucinations became prominent.  An FDG-PET, done 9 years after initial presentation and 18 months prior to death, is shown in Supplemental Figure 1B. There was significant bilateral caudate hypometabolism, but also prominent bilateral precuneus, posterior cingulate, parietal, temporal, and frontal hypometabolism. MRI was consistent with ventriculomegaly and is compared to a gross brain section in Supplemental Figure 2. This case represents multiple pathologies with iNPH potentially contributing early on. A brain autopsy revealed a brain weight of 1363g with a large cerebellar cyst, amyloid plaques and neurofibrillary tangles consistent with Braak Stage 4 AD, amyloid deposition in blood vessels consistent with amyloid angiopathy, and wide spread cortical and subcortical Lewy bodies consistent with DLB/PDD. This case highlights the importance of FDG-PET in assisting with diagnosis in complicated clinical presentations when multiple pathologies may be contributing.


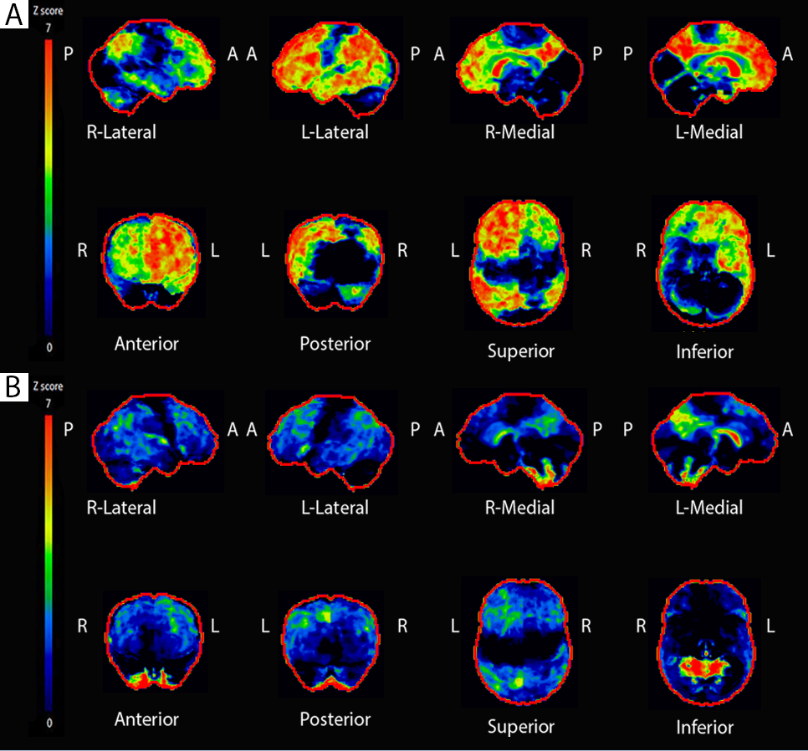


**Supplemental Figure 1:** FDG-PET scans in **A)** Patient 1 from the bvFTD group with a progranulin mutation resulting in diffuse severe hypometabolism including the L > R striatum. **B)** Patient 2 from the DLB/PDD group with multiple pathologic comorbidities including a large cerebellar cyst, Braak stage 4 Alzheimer’s disease, amyloid angiopathy, and diffuse cortical and subcortical Lewy bodies.


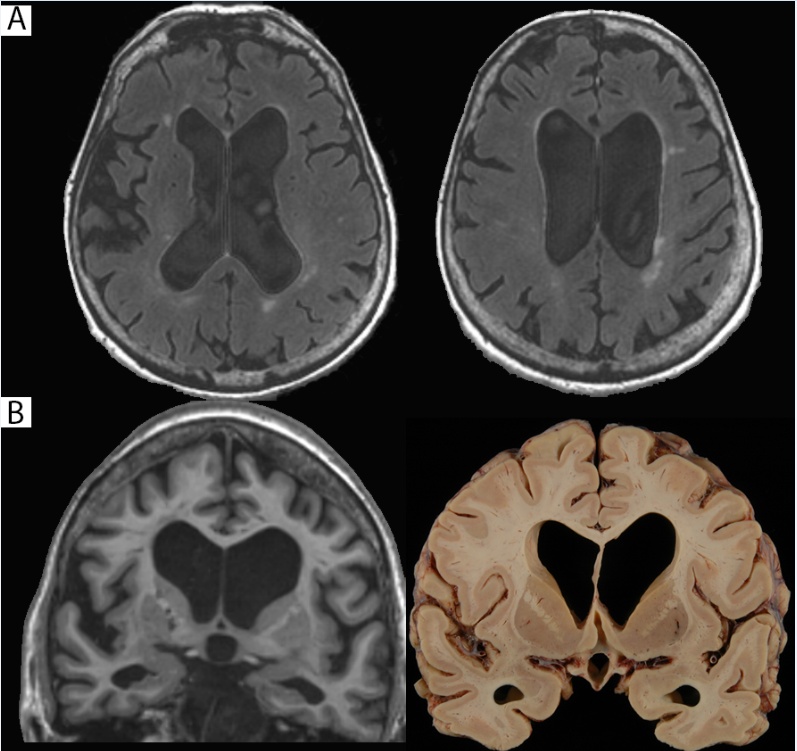


**Supplemental Figure 2:** Patient 2 from the DLB/PDD group showing: A) Axial MRI slices at initial presentation (10 years prior to death) showing significant ventriculomegaly. B) Coronal MRI slice 18 months prior to death matched with the autopsy gross specimen. The significant atrophy on MRI is present but diminished on the gross specimen, suggestive of contributing CSF dynamic alterations.
